# Mobile App Intervention on Reducing the Myeloproliferative Neoplasm Symptom Burden: Pilot Feasibility and Acceptability Study

**DOI:** 10.2196/33581

**Published:** 2022-03-31

**Authors:** Hninyee Win, Samantha Russell, Betsy C Wertheim, Victoria Maizes, Robert Crocker, Audrey J Brooks, Ruben Mesa, Jennifer Huberty, Holly Geyer, Ryan Eckert, Ashley Larsen, Krisstina Gowin

**Affiliations:** 1 Department of Medicine University of Arizona Tucson, AZ United States; 2 Cancer Center University of Arizona Tucson, AZ United States; 3 Andrew Weil Center for Integrative Medicine University of Arizona Tucson, AZ United States; 4 Mays Cancer Center University of Texas San Antonio, TX United States; 5 Department of Hematology University of Arizona Tucson, AZ United States

**Keywords:** myeloproliferative neoplasm, mobile application, symptom burden, wellness, self-management, mobile phone

## Abstract

**Background:**

Myeloproliferative neoplasms (MPNs) are a group of myeloid malignancies associated with significant symptom burden. Despite pharmacological advances in therapies, inadequate management of MPN symptoms results in reduced quality of life.

**Objective:**

This study aims to determine the feasibility of a 12-week global wellness mobile app intervention in decreasing MPN symptom burden. The University of Arizona Andrew Weil Center for Integrative Medicine’s global wellness mobile app, *My Wellness Coach* (MWC), guides patients to improve their health and well-being through facilitating behavior changes.

**Methods:**

Of the 30 patients enrolled in a 12-week intervention, 16 (53%) were retained through the final assessment. Feasibility was assessed by the ease of recruitment, participant adherence, and mobile app acceptability. App acceptability was measured using the user version of the Mobile Application Rating Scale. MPN symptom burden was measured at baseline and 12 weeks after the intervention.

**Results:**

Recruitment was efficient, with the participant goal reached within a 60-day period, suggestive of a demand for such an intervention. Adherence was less than the target within study design (75%), although similar to mobile device app use in other studies (53%). The app was deemed acceptable based on the mean user version of the Mobile Application Rating Scale 3-star rating by participants. Finally, there were statistically significant improvements in several MPN symptoms, quality of life, and total score on the Myeloproliferative Neoplasm Symptom Assessment Form surveys.

**Conclusions:**

Our 12-week intervention with the MWC app was feasible and was associated with a decrease in MPN symptom burden. Further investigation of the MWC app for use as a self-management strategy to reduce the symptom burden in patients with MPN is warranted.

## Introduction

### Background

Myeloproliferative neoplasms (MPNs) are a group of blood cancers and include the diagnoses of myelofibrosis, essential thrombocythemia, and polycythemia vera. These are rare malignancies with an incidence of 0.33, 1.14, and 1.18 per 100,000 persons per year, respectively [1-3]. Although the prevalence is relatively low, the chronicity of MPNs with significant disease-associated symptoms leads to many years of drastically reduced quality of life for patients afflicted with this disease. Disease manifestations vary among individuals, but generally, MPNs are characterized by excessive blood cells leading to an increased risk of blood clots or bleeding, enlargement of the spleen, and a significant symptom burden.

MPN symptom burden is heterogeneous and may be debilitating to afflicted patients. Fatigue is a major contributor to poor quality of life, affecting more than 80% of patients with MPN. Other symptoms include pruritus, night sweats, difficulty sleeping, abdominal discomfort, bone pain, fever, weight loss, decreased memory, poor sleep, inactivity, and psychosocial dysfunction [4-6]. Although the biology of symptoms is complex, it is rooted to the underlying physiologic inflammation associated with the disease and potentially contributing lifestyle factors and, thus, may be modifiable with intervention [7].

The discovery of the *JAK* *V617F* mutation in 2005 led to the subsequent development of JAK inhibitor therapy, which has revolutionized the treatment paradigm for MPNs. Despite improvements in disease outcomes offered by pharmacologic therapy, patients’ symptoms often remain quite high; thus, strategies to address symptoms represent a significant unmet need in the patient population of MPN [6].

Integrative oncology, defined as the use of complementary and integrative therapies in conjunction with conventional oncology care, may offer a unique symptom management tool [8,9]. Previous publications in integrative oncology have largely focused on breast, colon, and prostate cancers, and there is a scarcity of research to support integrative care in patients with MPN [6,10]. Nevertheless, early data using integrative approaches for the treatment of MPNs are promising, including aerobic activity, yoga, meditation, and strength training, to reduce the symptom burden and improve disease-related inflammation [6,11].

As thrombosis is the primary cause of mortality in MPNs, treatment is largely aimed at reducing thrombotic complications. In addition to pharmacologic treatments, lifestyle modifications through integrative methods may be an important tool in decreasing this risk as well. Inflammation is associated with increased risk of thrombotic events through various mechanisms, including elevated white blood cell and platelet counts, together with activated clotting factors and endothelial cells [12]. Regular exercise reduces inflammatory processes and promotes fibrinolytic activity [13,14]. Studies in patients with underlying cardiovascular disease that have established lifestyle change, including increased physical activity and the subsequent lowering of serum cholesterol, can decrease thrombosis risk [15]. It is possible that this same mechanism can decrease thrombotic risk in patients with MPN.

With the evolution of smartphone technology, mobile apps have become useful tools to help manage chronic diseases and may represent an important strategy for wellness-based interventions [16-24]. In oncology, mobile apps have largely focused on patient education and awareness, including such topics as cancer screening, adverse reactions to treatment, disease processes and treatment options, medication compliance, and cancer pain management [16,17,20,21,24,25]. A few mobile apps have emerged to address the symptom burden in endometrial, breast, sarcoma, and lung cancers with lifestyle changes, mindfulness, and coping mechanisms [18,22,23]. Although smartphone-based meditation and web-based yoga have proven some benefit in patients with MPN, with early data specifically suggesting impact on fatigue, depression, and blood inflammation, there has not been a mobile app to specifically address the MPN symptom burden through promoting global behavioral changes [19,26].

### Objectives

The University of Arizona Andrew Weil Center for Integrative Medicine has recently developed and successfully piloted a global wellness mobile app, *My Wellness Coach* (MWC), to guide patients to better health and wellness through facilitating behavior changes [27]. The aim of this study is to determine the feasibility of a global wellness mobile app 12-week intervention for decreasing MPN symptom burden. This is meaningful because this app could provide a highly disseminatable and much needed self-management strategy for the MPN symptom burden.

## Methods

### Ethics Approval

This study was approved by the University of Arizona Human Subjects Protection Program (IRB Number 2004567060).

### Recruitment and Enrollment

Patients with MPN were recruited nationally through organizational partners, such as the Myeloproliferative Neoplasm Quality of Life Group. A flyer was posted on the partners’ website, and an email describing the study was sent out through approved listservs, with a link to a web-based eligibility consent and survey over a 60-day period. Through this web-based survey, developed in Qualtrics [28], participants consented and answered questions regarding the eligibility requirements of the study. Participants were screened for eligibility using the inclusion and exclusion criteria given in Textbox 1.

Once a web-based questionnaire was returned, it was evaluated for completeness and eligibility based on the responses. A participant was then emailed the consent form and the baseline questionnaire that were required for enrollment in the study. These participants were given approximately 3 days to respond. If they did not respond within the allocated time, then they were deemed as not interested in participating in the study, and the baseline questionnaire was sent to the next eligible participant. Eligible participants were contacted until our enrollment goal of 30 individuals was met. An optional phone interview was conducted for qualitative analysis at 12 weeks.

**Inclusion and exclusion criteria.**

**Inclusion criteria**

- Individuals, man or woman, aged ≥18 years
- Individuals able to provide consent
- Individuals who can speak English and is English literate
- Individuals diagnosed of essential thrombocythemia, polycythemia vera, or myelofibrosis
- Individuals identified by a treating physician
- Individuals who have a personal mobile device with an Android (version 4.1 or higher) or iOS (version 12.2 or higher) operating system
- Individuals who displayed satisfactory ability to operate a mobile device and showcase email communication
- Individuals who have reliable internet and cellular access

**Exclusion criteria**

- Individuals currently participating in a clinical trial
- Individuals who received blood transfusion within the last 6 months
- Individuals with an Eastern Cooperative Oncology Group [29] status of ≥3

### Mobile App Intervention

After consent was obtained from eligible participants, they were provided with instructions to download and use MWC. In the app, the participants were prompted to participate in four steps: (1) score, (2) explore, (3) set a goal, and (4) take action. Self-motivation was emphasized by encouraging reflection on why they want to facilitate change. Clinical research staff suggested setting at least two wellness goals. Participants could set goals within seven domains: nutrition, movement, sleep, resilience, environment, relationships, and spirituality. The app provided tips for health improvements and links to curated resources. When setting goals, participants were coached to make them *SMART*—specific, measurable, attainable, relevant, and time-bound—with the value of this technique described in other publications [30-32]. It provided 24- to 72-hour interval reminders before and after each action step and a goal deadline to encourage action throughout the intervention.

### Outcome Measures

Feasibility was assessed by ease of recruitment, adherence, and mobile app rating. Successful recruitment was defined as enrolling 30 participants within 60 days. Adherence was defined as a minimum of 75% retention throughout the intervention. The user version of the Mobile Application Rating Scale (uMARS) evaluated acceptability through rankings in four objective categories (engagement, functionality, aesthetics, and information) and was graded on a Likert scale. This included ease of use, intent for continued use, perceived benefit, and overall star rating.

The Myeloproliferative Neoplasm Symptom Assessment Form (MPN-SAF) evaluated common MPN symptoms [33]. The MPN-SAF comprises 18 symptoms plus a total score. Higher scores indicate worse symptom severity. Each symptom was rated according to the level of difficulty a participant had with each item during the past week, scaled from 0 (absent) to 10 (worst imaginable).

The Behavioral Risk Factor Surveillance System (BRFSS) was used to assess physical activity [34]. The 8-item Patient-Reported Outcomes Information System (PROMIS) Sleep Disturbance questionnaire (Short Form 8a) measured perceptions of sleep quality, depth, and restoration within the past 7 days [35]. Mediterranean diet adherence was assessed using a 14-item questionnaire (which has previously shown an inverse association between the Mediterranean diet and obesity in the PREDIMED [Prevención con Dieta Mediterránea] trial [36]). However, the original 14-point scale was revised because 40% of participants were unable to quantify olive oil consumption and responded “don’t know/not sure” or left responses blank. At 12-week follow-up, MPN-SAF, BRFSS, PROMIS Sleep, and Mediterranean diet adherence questionnaires were completed in addition to uMARS. Semistructured phone interviews with the research coordinator to provide additional feedback were optional.

### Statistical Analysis

To assess recruitment, acceptability, length of time to accrue, adherence, ease of app use, intent for continued use, and perceived benefit measures were summarized using descriptive statistics. Patient demographics and medical history were summarized using descriptive statistics. Descriptive statistics included proportions for categorical variables and median (IQR) or mean (SD) for continuous variables. Mean changes from baseline to 12 weeks in sleep disturbance, Mediterranean diet adherence, and MPN-SAF symptom scores were tested using linear mixed-effects models, clustered on participants. Such mixed-effects models were used instead of 2-tailed paired *t* tests to allow all participants (n=30) to contribute to the model, instead of only the subset (n=16) with the end-of-study data. The *α* level was set to .05, meaning that mean values at 12 weeks were considered significantly different from the mean values at baseline if *P*<.05. All statistical analyses were conducted using Stata (version 16.1; StataCorp). As this is a feasibility study, there were no adjustments made for multiple comparisons because of the small sample size.

## Results

### Recruitment and Enrollment

**Figure 1:**
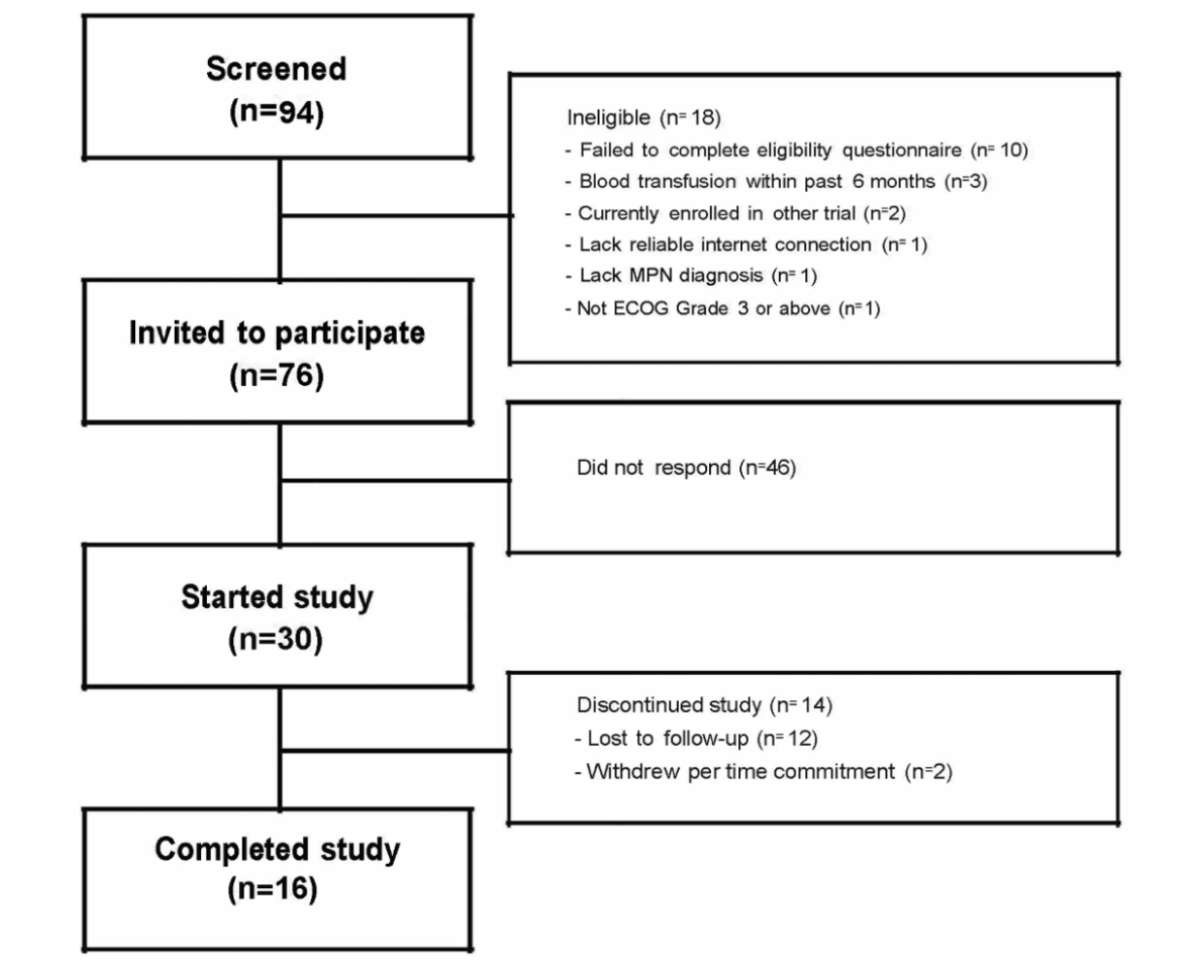
Outline of participant enrollment. ECOG: Eastern Cooperative Oncology Group; MPN: myeloproliferative neoplasm.

Of the 94 interested patients with MPN screened for eligibility, 72 (76%) were eligible to enroll (Figure 1). Emails were sent to eligible patients until 30 were successfully enrolled, which was accomplished within a 3-week period.

### Patient Demographics

The median age of participants was 62.6 (IQR 54.1-70.2; mean 60.5, SD 12.6) years, and participants were predominantly women (26/30, 87%) and White (28/30, 93%). MPN diagnoses included essential thrombocythemia (11/30, 37%), polycythemia vera (11/30, 37%), and myelofibrosis (8/30, 27%). Most participants (23/30, 76%) were diagnosed with MPN >5 years ago. Of the 30 participants, 11 (37%) reported a known history of splenomegaly, 12 (40%) documented a previous diagnosis of anemia, and 6 (20%) reported a history of blood clots after being diagnosed with MPN. Most participants were being treated with hydroxyurea (20/30, 67%), and some (8/30, 27%) were being treated with ruxolitinib or another JAK inhibitor. Of the 12 participants who completed questions pertaining to the Dynamic International Prognostic Scoring system for myelofibrosis, 1 (8%) was in the high-risk group and 6 (50%) were in the intermediate-1–risk group. There were no statistically significant differences in any of the aforementioned characteristics between participants who were retained in the study (16/30, 53%) and those who were not (14/30, 47%; Table 1).

**Table 1:** Baseline characteristics of participants.

Characteristic		All participants (n=30)	Retained participants (n=16)
Age (years), median (IQR)		62 (54.1-70.2)	62 (54.9-67.5)
BMI (kg/m^2^), median (IQR)		24.5 (21.9-29.8)	23 (20.8-34.6)
**Gender, n (%)**			
	Man	3 (10)	2 (13)
	Woman	26 (87)	13 (81)
	Prefer not to answer	1 (3)	1 (6)
**Race, n (%)**			
	Asian population	1 (3)	1 (6)
	White population	28 (93)	15 (94)
	Prefer not to identify	1 (3)	0 (0)
**Ethnicity, n (%)**			
	Non-Hispanic or Latino(a) population	25 (96)	14 (100)
	Prefer not to identify	1 (4)	0 (0)
**Language, n (%)**			
	English	30 (100)	16 (100)
**MPN^a^ diagnosis, n (%)**			
	Essential thrombocytosis	11 (37)	7 (44)
	Polycythemia vera	11 (37)	5 (31)
	Myelofibrosis	8 (27)	4 (25)
**Year of MPN diagnosis, n (%)**			
	2015 to present	7 (24)	4 (27)
	2010-2014	13 (45)	6 (40)
	Before 2010	9 (31)	5 (33)
History of enlarged spleen, n (%)		11 (37)	6 (38)
History of anemia, n (%)		12 (40)	4 (25)

^a^MPN: myeloproliferative neoplasm.

### MWC Mobile App Use and Rating

Of the 30 enrolled participants, 7 (23%) failed to make an MWC account, and 10 (33%) additional participants did not set wellness goals within the app. Of the remaining 13 participants, 10 (33%) set at least one or two goals (a total of 5 goals allowed). The uMARS questionnaire was successfully completed by 43% (13/30) of the participants. Most categories had a mean score at ≥3 (Table 2). The highest rated category was aesthetics (mean score 3.4, SD 0.8), and the lowest rated category was behavior change (mean score 2.9, SD 1.2). Most participants (7/13, 54%) gave the MWC app an overall 3-star rating acceptable on the 5-point scale [37].

A total of 6 participants provided additional feedback in an optional phone interview. Participants praised that the app addressed “every aspect of healthy living” and found it resourceful. In addition, 3 participants stated that the app encouraged them to work toward achieving their goals. Then, 1 participant specified that it helped her recognize “that there are definite areas that I need to work on and it helped to get a good start working in the right direction.”

Most participants (5/6, 83%) felt that additional guidance in setting up goals would be helpful. Other feedback included the frequency of the reminders seemed “too much” and the tone of the reminders could potentially be adjusted.

**Table 2:** The user version of the Mobile Application Rating Scale (n=13).

Rating		Values
**Category, mean (SD; range)**		
	Engagement	3.0 (1.1; 1.0-5.0)
	Functionality	3.2 (1.3; 1.0-5.0)
	Aesthetics	3.4 (0.8; 2.0-5.0)
	Information	3.2 (1.4; 1.0-5.0)
	Overall quality	3.1 (1.1; 1.0-5.0)
	Awareness	3.2 (1.3; 1.0-5.0)
	Knowledge	3.2 (1.4; 1.0-5.0)
	Attitudes	3.2 (1.3; 1.0-5.0)
	Intention to change	3.2 (1.4; 1.0-5.0)
	Help seeking	3.0 (1.5; 1.0-5.0)
	Behavior change	2.9 (1.2; 1.0-5.0)
**Overall star rating (single question), n (%)**		
	1 star	2 (15)
	2 stars	2 (15)
	3 stars	7 (54)
	4 stars	1 (8)
	5 stars	1 (8)

### Adherence and Acceptability

All 30 participants were successfully enrolled within the allotted 60-day period. Only 53% (16/30) of participants were retained throughout the 12-week study. Attrition was due to either voluntary withdrawal from the study (2/30, 7%) or loss to follow-up for unknown reasons (12/30, 40%). On the basis of the uMARS score as outlined in the *MWC Mobile App Use and Rating* section, MWC was rated a mean score at ≥3 in many categories, with an overall 3-star rating by most of the participants. The baseline characteristics of participants who completed the study and those who did not were compared, and no statistically significant differences were found.

### Patient-Reported Outcomes

The MPN-SAF responses highlighted that not all symptoms were changed by the 12-week intervention, but several (8/18, 44%) significantly improved. Improvements were observed in inactivity, impaired concentration, dizziness, numbness, sexual dysfunction, night sweats, bone pain, and quality of life (Table 3). Furthermore, the mean total score was significantly better at 12 weeks than at baseline (mean 54.8, SD 31.6, vs mean 68.4, SD 30.5; *P*=.001).

The PROMIS Sleep Disturbance (Short Form 8a) responses suggested that mean sleep disturbance scores did not significantly change between baseline (mean 24.2, SD 7.2) and at 12 weeks (mean 22.2, SD 7.4; *P*=.49). Few participants reported moderate or severe sleep disturbance at baseline (5/30, 17%) or 12 weeks (3/16, 19%; Table 4). Furthermore, there was a marginally significant (*P*=.06) difference in Mediterranean diet adherence between baseline (mean 5.9, SD 2.3) and at 12 weeks (mean 6.2, SD 2.7).

Of the 16 participants who completed the BRFSS survey, 16 (100%) responded *yes* to *any physical activities or exercises* at baseline compared with 13 (81%) at 12 weeks. The most frequent types of physical activity or exercise included walking or hiking (8/16, 50% participants at baseline; 7/16, 44% at 12 weeks), yoga, Pilates, or barre (3/16, 19% at baseline; 2/16, 13% at 12 weeks), and gardening (2/16, 13% at baseline; 0/16, 0% at 12 weeks). Participants generally reported a high frequency of engagement in their top physical activity, with most participants reporting 5 to 6 times per week (6/16, 38% at baseline; 6/16, 38% at 12 weeks) or ≥7 times per week (4/16, 25% at baseline; 4/16, 25% at 12 weeks).

**Table 3:** Assessment of symptom severity based on MPN-SAF using a mixed-effects model.

Symptom	Baseline (n=30), mean (SD)	12 weeks (n=16), mean (SD)	*β* coefficient	*P* value
Early satiety	4.5 (2.9)	3.8 (3.1)	−0.59	.21
Abdominal pain	2.6 (2.5)	2.8 (2.9)	.10	.80
Abdominal discomfort	3.5 (2.8)	3.6 (3.1)	.01	.99
Inactivity	5.2 (2.6)	3.9 (2.8)	−1.18	.02
Headaches	3.6 (3.0)	2.9 (1.9)	−0.50	.33
Impaired concentration	5.2 (3.0)	3.6 (2.6)	−1.13	.03
Dizziness	4.0 (2.8)	3.1 (2.4)	−1.07	.02
Numbness	3.8 (2.6)	3.0 (2.0)	−0.96	.02
Insomnia	5.9 (3.4)	4.8 (3.1)	−0.53	.20
Depression	3.5 (2.6)	3.2 (2.5)	−0.04	.93
Sexual dysfunction	5.2 (3.8)	3.9 (3.5)	−1.62	.01
Cough	1.9 (1.6)	1.9 (1.9)	−0.23	.50
Night sweats	3.7 (2.5)	2.7 (1.9)	−1.01	.001
Itching	3.7 (3.2)	2.8 (2.7)	−0.05	.84
Bone pain	3.3 (2.8)	2.4 (2.2)	−0.71	.02
Fever	1.3 (1.0)	1.1 (0.2)	.05	.37
Weight loss	2.2 (2.5)	1.4 (0.9)	−0.70	.10
Quality of life	5.3 (2.6)	3.8 (2.2)	−1.27	.03
MPN-SAF^a^ total score	68.4 (30.5)	54.8 (31.6)	−11.19	.001

^a^MPN-SAF: Myeloproliferative Neoplasm Symptom Assessment Form.

**Table 4:** Sleep disturbance based on the Patient-Reported Outcomes Information System.

Level	Baseline (n=29), n (%)	12 weeks (n=16), n (%)
None to slight	14 (48)	10 (63)
Mild	10 (34)	3 (19)
Moderate	4 (14)	3 (19)
Severe	1 (3)	0 (0)

## Discussion

### Principal Findings

There is a high demand in the patient population of MPN for innovative interventions to help alleviate their symptom burden [4,38]. The aim of this study is to determine the feasibility of a 12-week global wellness mobile-based intervention to reduce symptom burden in patients with MPN. This is the first known study to use a global wellness mobile app to address the symptom burden of patients with MPN through advocating self-management strategies focused on facilitating behavioral change. Our results suggest that this study is feasible in terms of the successful enrollment of 30 MPN participants within 30 days and mobile app acceptability by patients. Previous publications, with many recent additions in the medical literature over the last 2 years, have suggested that mobile health apps provide a feasible option to improve quality of life in various patients with cancer, especially through self-care support and behavior modification [22,23,39-41]. However, no mobile health apps have yet to be tested in the patient population of MPN. Most smartphone apps suggest benefit in symptom management; however, none, to our knowledge, have incorporated symptom management and global lifestyle change within 1 app. In small pilot studies, a smartphone-based meditation (CALM) and web-based yoga have previously been shown to be feasible in the population with MPN, with suggested impact on fatigue, sleep, pain, anxiety, and depression [19,26]. Although these pilot studies suggest benefit in symptom management with an isolated behavior intervention (ie, meditation or yoga), the app tested in our study focused on the feasibility of a global lifestyle intervention. A pilot study of MWC showed similar successful recruitment of patients and simple delivery of a nonpharmacologic intervention [27]. The ease and timeliness of this process suggest that this app may be an effective method for providing a self-management strategy for symptom burden in patients with MPN that is highly disseminable to wide groups of individuals.

With most uMARS mean scores at ≥3 points (of a total possible 5), the mobile app was deemed acceptable to this small group of participants [42]. The lowest rated category was behavior change, with a lower mean score of 2.9, close to the mean score in previous studies. Comparatively, the highest rated category was aesthetics, with a mean score of 3.4. The app could potentially be improved by addressing feedback from participants who stated that the mobile app would benefit from additional information describing the process for goal setting, as well as some technical glitches. Furthermore, our study did not achieve the 75% defined target for adherence, with only 53% (16/30) of the participants retained through the 12-week final assessment. However, this finding is quite common; other studies using mobile device health apps note that most users stop use soon after initial use [43] and that approximately half of the 934 survey responders (45.7%) had stopped using a mobile health app [41]. Another study revealed that only 54% of individuals who download a mobile device health app persisted in using it >1 month [44]. Of the 47% who did not complete the final assessment, most participants were lost to follow-up for unknown causes. Retention in future studies could potentially be improved by having more communication with study coordinators throughout the 12-week duration of the intervention check in and assist with any technical difficulties or questions [45].

MWC aims to provide guidance and facilitate wellness goals. By comparing baseline MPN-SAF assessments with 12-week evaluations, improvement was noted in the areas of inactivity, impaired concentration, dizziness, numbness, sexual dysfunction, night sweats, bone pain, and quality of life. Furthermore, the total score improved after the intervention. Thus, the 12-week intervention with MWC suggests a positive impact on our small study group, with improvement in the overall symptom burden. An app feature that may have contributed to the improvement of the symptom burden is the reminder to act on goals. Previous studies have shown that apps that remind participants have better use rate than apps that did not [44,46]. Given our small group of participants, larger studies will be needed to validate the true impact of MWC on MPN symptoms.

The mobile app’s impact on diet did not show significant statistical improvement in Mediterranean diet adherence after the 12-week intervention. Limitations in scoring for Mediterranean diet adherence was noted because of participants leaving certain questions blank or responding as “don’t know/not sure.” In addition, regarding physical activity, the BRFSS survey results were limited in assessing the quantitative change using statistical methods. Therefore, future studies may need to determine a different method for evaluating physical activity that allows for scoring of physical activity. However, participants were noted to engage in their physical activity of choice at high frequency, with 10 participants engaging 5 to 6 times per week or more. However, the impact on sleep was not statistically significant. Although lifestyle changes were not significantly improved in this pilot study, future analysis with a larger patient sample and potentially alternate dietary and physical activity measures may improve the study design. Poor cardiometabolic health is a primary contributor to mortality in patients with MPN, and effective lifestyle interventions and continued study of lifestyle interventions are needed.

Overall, the study exhibited ease of recruitment and acceptability, and although adherence was lower than our goal, it was commensurate with other mobile health app interventions. Finally, the 12-week mobile app pilot study improved the MPN symptom burden and warrants continued study with a larger sample size.

### Limitations

The most notable limitation to our study was the small study size. Not achieving the defined level of adherence is a concern; however, the 75% target was likely set too high when compared with the mobile health app use found in the literature and, therefore, may point to a limitation of study design [41,43]. In addition, only 53% (16/30) of the participants completed the study, and 47% (14/30) voluntarily withdrew or were lost to follow-up. On the basis of the completion results in this study, future studies could potentially increase the recruitment goal to ensure a larger sample completing the intervention.

Additional limitations of the study include variability in the proficiency of modern technology by the participants and inability to collect data regarding weekly use of the app. Some participants admitted to discontinuing the mobile app because of confusion and difficulty in understanding how to properly use the app. This limitation could be surmounted by future study design, including frequent coordinator and group engagement for app support. Participants also expressed concerns regarding the frequency of reminders, with a participant stating that it was too frequent and another participant stating that she was not reminded enough. These limitations highlight the importance of a user-friendly mobile app platform with the ability for customization. Perhaps letting the users set their own frequency for reminders can improve adherence. In addition, the study was conducted during the COVID-19 pandemic, when gym closures, self-isolation, and mental health issues could confound the participants’ sleep, diet, and physical activity. A participant specifically stated that she “would have used the app for better success if [she] hadn’t been self-isolating during this pandemic.”

Access barriers, such as patients with MPN who do not possess a mobile device, reliable internet connection, or limited literacy, should also be acknowledged. Although no adaptations for the older patient population were incorporated in this feasibility study, users have the ability within the app to increase the font size on their mobile device for visual limitations. In addition, users can dictate narrative responses (voice-to-text functionality), which can reduce the typing required. App adaptations for the older population will be considered in future design and study of the mobile app. Finally, this study did not investigate which mechanisms were most effective at reducing specific symptoms, as this would require a much larger sample size, and this should be explored in future studies.

### Conclusions

Our small feasibility pilot study provided preliminary evidence that the MWC global wellness app intervention is feasible within the population with MPN and may reduce the MPN symptom burden. There was insufficient evidence to show improvements in physical activity vigor and frequency, healthy diet consumption, and better sleep quality. Future trials evaluating the impact of the MWC mobile app in the population with MPN could be strengthened by a larger sample size, measures to improve adherence and retention (such as coordinator support), and closer consideration of diet and physical activity measures. Overall, this study provides a promising first step toward a self-management strategy to lessen the substantial symptom burden of patients with MPN.

## Abbreviations

BRFSS: Behavioral Risk Factor Surveillance System
MPN: myeloproliferative neoplasms
MPN-SAF: Myeloproliferative Neoplasm Symptom Assessment Form
MWC: My Wellness Coach
PREDIMED: Prevención con Dieta Mediterránea
PROMIS: Patient-Reported Outcomes Information System
uMARS: user version of the Mobile Application Rating Scale
